# Clinical impact of continuous nursing under IMB mode combined with nutrition intervention on body composition, nutritional indicators, and negative emotions of patients undergoing metabolic bariatric surgery

**DOI:** 10.3389/fsurg.2025.1676904

**Published:** 2025-11-10

**Authors:** Lingling Zhou, Yuan Xia, Yanyu Qiu, Yangyang Yao, Jinsheng Wu, Guangnian Ji, Cui Liu

**Affiliations:** Department of Hepatobiliary and Pancreatic Surgery, The Affiliated Huai’an No.1 People’s Hospital of Nanjing Medical University, Huai’an, Jiangsu, China

**Keywords:** obesity, metabolic bariatric surgery, IMB, continuous nursing, body composition, nutrition intervention

## Abstract

**Aim:**

The research aimed to elucidate application impact of continuous nursing under IMB mode combined with nutrition intervention on patients after metabolic bariatric surgery (MBS).

**Methods:**

Eighty obese patients who underwent MBS in our hospital from January 2021 to January 2023 were divided into a control group (CG, *n* = 40) and an experimental group (EG, *n* = 40). The CG received conventional continuous nursing, while the EG received continuous nursing under the IMB mode combined with nutrition intervention. Body composition, biochemical indicators, and negative emotion scores were compared between the two groups before and after surgery.

**Results:**

One month and three months after surgery, weight, fat-free mass, muscle mass, fat mass, visceral fat area, and total cellular water content in both groups demonstrated depletion relative to those before surgery (*P* < 0.05), whereas fat-free mass and muscle mass in EG demonstrated elevation relative to those in CG during the same period (*P* < 0.05), and no statistical significance in weight, fat mass (FM), visceral fat area, and total cellular water content demonstrated between both groups during the same period (*P* > 0.05); C-reactive protein (CRP), and glycated hemoglobin (HbA1c), levels in both groups demonstrated depletion relative to those before surgery (*P* < 0.05), and CRP and HbA1c levels in EG demonstrated depletion relative to those in CG during the same period (*P* < 0.05); serum albumin (ALB), prealbumin (PA), vitamin A, vitamin B_1_, vitamin B_2_, vitamin B_6_, vitamin C, and ferritin levels in both groups demonstrated depletion relative to those before surgery (*P* < 0.05), whereas ALB, PA, vitamin A, vitamin B_1_, vitamin B_2_, vitamin B_6_, vitamin C, and ferritin levels in EG demonstrated elevation relative to those in CG during the same period (*P* < 0.05); Hamilton Anxiety Scale (HAMA) and Hamilton Depression Scale (HAMD) scores in both groups demonstrated depletion relative to those before surgery, and HAMA and HAMD scores in EG demonstrated depletion relative to those in CG during the same period (*P* < 0.05).

**Conclusion:**

Combining IMB-based continuous nursing and nutrition intervention reduces post-MBS anxiety and depression, preserves muscle mass, improves nutritional status, and enhances public acceptance of MBS, promoting its advancement.

## Introduction

Obesity is gradually becoming a global public health issue. The occurrence of obesity has relation to consuming high calorie and processed foods, as well as a lack of physical activity (1, 2). Obesity increases the risk of type 2 diabetes mellitus (T2DM), cardiovascular diseases and tumors, and also enhances social and economic burden.

Metabolic bariatric surgery (MBS) is currently one of the long-term effective treatment measures for severe obesity (3). The commonly applied methods in clinical practice are sleeve gastrectomy (SG) and Roux-en-Y gastric bypass (RYGB) (4). In China, patients with body mass index (BMI) ≥ 40 kg/m^2^ or BMI of 27.5–39.9 kg/m^2^ with at least one comorbidity [such as T2DM, hyperlipidemia, etc.] are defined as indications for MBS (5, 6). The purpose of MBS is to control weight gain through reducing calorie intake; according to mechanism of reducing calorie intake, MBS can receive classification into two categories: restriction and malabsorption (7). The reduction in absorption area and restricted food intake after MBS may lead to nutritional deficiencies. All MBS surgeries can elevate risk of postoperative micronutrient deficiency in patients, and risk of micronutrient deficiency due to malabsorptive surgery is more severe relative to that of restrictive surgery (8). Thus, postoperative medical nutritional therapy is particularly crucial. In addition to physical challenges, patients who undergo MBS surgery also face significant psychological adjustment pressures. Research indicates that some patients may experience anxiety, depression, or body image concerns following surgery. These negative emotions may stem from worries about weight regain and changes in eating habits (9, 10). Without timely intervention, such feelings can compromise adherence to dietary and nutritional plans, thereby undermining the long-term success of the procedure. Therefore, comprehensive postoperative care protocols must prioritize patients’ mental health.

Continuous nursing is an orderly, coordinated, and uninterrupted professional and informal treatment and care behavior provided for patients in different health service systems or under different conditions of the same health service system to meet their actual needs after hospitalization (11). Currently, continuous nursing services mostly adopt telephone follow-up and home visits, majorly relying on one-way output from nursing staff, which makes it difficult to help patients establish behavioral motivation changes, resulting in patients being unable to adhere to healthy behaviors in a long term. The Information-Motivation-Behavioral-Skills (IMB) model is a behavioral change theory proposed by Fisher et al., which makes it easier for patients to internalize into practice and improve long-term prognosis (12, 13). Nursing interventions based on the IMB model have demonstrated progress in managing certain chronic diseases. For instance, it effectively increases the frequency of self-monitoring of blood glucose among diabetic patients, improves dietary choices, and promotes regular exercise (14, 15). The IMB model also serves as a key theoretical framework for HIV prevention and management (16, 17). Therefore, we have applied the IMB model to post-operative care following MBS procedures.

The research aimed to elucidate application impact of continuous nursing under IMB mode combined with nutrition intervention on patients after MBS, which may provide new ideas for seeking out-of-hospital nursing work suitable for patients after MBS.

## Materials and methods

### General data

Eighty obese patients who underwent MBS in our hospital from January 2021 to January 2023 received selection and random division into a control group (CG) and an experimental group (EG) with a random drawing method, with 40 cases each. Inclusion criteria: 1) Age ranging 18–65 years old; BMI ≥ 35 kg/m^2^; female waist circumference >85 cm, male waist circumference >90 cm; 2) difficulty in controlling weight through adjusting diet and physical exercise, or accompanied by comorbidities; 3) able to tolerate surgery with decent postoperative recovery, without severe heart, lung, kidney and other diseases; 4) those without a history of mental illness, possessing certain self-care abilities, and able to effectively cooperate with this research; 5) patients and their family members were informed and voluntarily participated in this research. Exclusion criteria: 1) Those with primary diseases such as liver and kidney systems, hematopoietic systems, cerebrovascular systems, and severe endocrine disorders requiring long-term medication; 2) patients and their family members with low education level and unable to complete questionnaire survey, or who may withdraw midway and cannot obtain follow-up results. All participants signed informed consent, and this research received approval by the medical ethics committee of our hospital.

This investigation utilized a prospective, randomized controlled trial methodology. Eighty participants were recruited and allocated to the experimental or control group in a 1:1 ratio through a computer-generated randomization sequence. Allocation concealment was ensured using sequentially numbered, opaque, sealed envelopes. Although it was not possible to blind patients and healthcare providers administering the treatment, measurement bias was minimized by keeping the researchers who collected outcome data—particularly blood biochemical markers and negative emotion scores—blinded to group assignments and uninvolved in patient care. Additionally, there were no participant withdrawals or losses to follow-up during the study.

### Methods

During patients’ hospitalization, after completing relevant examinations, nursing staff should adopt symptomatic treatment based on postoperative conditions of both groups, supplemented by health education such as diet, exercise, medication, self-management, etc. The CG received conventional continuous nursing, including postoperative follow-up, routine dietary guidance, exercise guidance, and management of common complications. The OG implemented continuous nursing under IMB mode combined with nutrition intervention on the basis of CG.

An IMB team consisting of one co-chief superintendent nurse, one supervising nurse, and one psychological counselor, as well as six responsible nurses should receive establishment; co-chief superintendent nurse should serve as team leader, majorly responsible for overall planning and arrangement; psychological counselor should provide training on IMB related skill models and skills; supervising nurse should be responsible for collection of research data; relevant responsible nurses should be responsible for implementation of continuous nursing.

Information intervention: The IMB team will independently develop a *Postoperative Nursing Manual for Metabolic Bariatric Surgery*, which includes knowledge related to obesity, MBS, diet and lifestyle, adverse reactions and prevention, as well as psychological and rehabilitation guidance. The manual will inform patients of necessity of scheduled follow-up examinations and serve as a basis for providing information support to patients. Nursing staff should create a WeChat official account and set up five knowledge modules in WeChat official account, including disease knowledge education, diet management, exercise guidance, medication guidance, and self-management; moreover, nursing staff should add patients’ WeChat to patient communication group; thus patients can obtain disease related knowledge through WeChat official account and WeChat patient communication group.

Motivation intervention: Before discharge, nursing staff should conduct motivational interviews to understand psychological needs of patients, explain importance of healthy behaviors in obesity treatment, and guide patients to cultivate health awareness; in WeChat patient communication group, nursing staff should encourage patients to discuss and share successful experiences with each other.

Behavioral intervention: Outpatient follow-up reminder: Nursing staff should send information through Health Manager platform of hospital in the 1st, 2nd, and 3rd months after patients are discharged, reminding patients to come to hospital for subsequent visit on time every month. Self-management behavior tracking: Nursing staff should require patients to record implementation of their self-management plan daily after discharge and check in in WeChat patient communication group. At the 1st, 2nd, and 3rd months after discharge, nursing staff should conduct telephone follow-up visits on patients’ diet, exercise, medication, and other conditions, and conduct one-on-one personalized behavioral evaluations and guidance.

Nutritional intervention: All patients received nutritional guidance based on standard postoperative metabolic weight loss surgery guidelines. However, the OG group received a more structured, phased, and goal-oriented nutritional enhancement management program, differing from the CG's routine recommendations as outlined below.

Nursing staff should develop nutritional therapy for six stages in three months after MBS in accordance with the *Consensus on Nutritional and Multi-disciplinary Management for Metabolic Bariatric Surgery* in 2018 (18) and the *Clinical Practice Guidelines for the Perioperative Nutritional, Metabolic, and Nonsurgical Support of the Bariatric Surgery Patient–2013 Update* in the USA (19). 1) 1 day after surgery: Fasting and water deprivation; 2) 2–3 days after surgery: drinking water; 3) 4–6 days after surgery: water + whey protein powder ≥45 g/d; 4) 1–2 weeks after surgery: fluid + whey protein powder of 45 g/d, protein 40–60 g/d, an energy intake of 42 kJ/(kg · d) (ideal weight); 5) 3–4 weeks after surgery: a semi-liquid diet, protein 60–80 g/day, an energy intake of 63 kJ/(kg · d) (ideal weight); 6) 2–3 months after surgery: Establish sustainable balanced dietary patterns to prevent malnutrition, protein ≥80 g/day, energy intake of 84 kJ/(kg · d) (ideal weight). Some patients may experience anemia, hair loss, and muscle twitching in early postoperative period. Nursing staff should guide patients to adjust dietary habits and structure and take appropriate measures such as supplementing vitamins and microelements to effectively alleviate symptoms; nursing staff should advise patients to regularly review clinical indicators such as hemoglobin, vitamin D, blood calcium, vitamin B_12_, folic acid, serum iron, 25 hydroxyvitamin D, parathyroid hormone, etc. For patients in the CG group, care primarily relies on routine support, involving standard health education and verbal encouragement provided by physicians or nurses during follow-up visits. The OG group receives enhanced support based on the IMB model, addressing adherence through three dimensions: information, motivation, and behavioral skills.

### Observation indicators

#### Primary outcome measure

1. Body composition indicators: Before, 1 month and 3 months after surgery, body composition received measurement using a multi-frequency and multi-contact body composition analyzer (Inbody770; Biospace; Korea). The fat free mass (FFM), muscle mass, fat mass (FM), visceral fat area, total cellular water content, etc., in both groups received recording. The above indicators received measurement on an empty stomach at 8 o'clock in the morning. Compare the general characteristics of the two patient groups based on the above indicators.
2. Biochemical indicators: Before, 1 month and 3 months after surgery, venous blood samples received collection from patients for measuring C-reactive protein (CRP), glycated hemoglobin (HbA1c), serum albumin (ALB), prealbumin (PA), as well as vitamins A, B_1_, B_2_, B_6_, C, and ferritin levels in both groups. Compare the inflammatory, glycemic, and nutritional indicators between the two patient groups based on the above metrics.

#### Secondary outcome measures

1. Negative emotion scores: The anxiety and depression scores in both groups before and 1, 3, 6, 9 and 12 months after surgery received observation and recording. Evaluation was conducted using the Hamilton Anxiety Scale (HAMA) and Hamilton Depression Scale (HAMD) (20). All items are scored through a 5-point rating system ranging 0–4, with 0 for asymptomatic symptoms, 1 for mild symptoms, 2 for moderate symptoms, 3 for severe symptoms, and 4 for critical symptoms. The higher the scores, the more severe the patients’ anxiety and depression symptoms. Compare the negative emotion scores between the two groups of patients based on the above indicators.

### Statistical analysis

This research adopted SPSS 27.0 software for data statistical analysis. Counting data received description in (%), followed by *χ*^2^ test for intergroup comparisons. Quantitative data conforming to a normal distribution received description using mean ± standard deviation (x ± s), followed by repeated-measures analysis of variance to test for intergroup differences. *post hoc* tests were performed for pairwise comparisons within groups (compared to baseline) and between groups (at the same time point), with Bonferroni correction applied for multiple comparisons. The difference was statistically significant with *P* < 0.05.

## Results

### Comparison of general data between both groups

The general data between both groups demonstrated no statistical significance (*P* > 0.05; Table 1), indicating comparability.

**Table 1:** General data in both groups.

General data		Control group (CG) (*n*=40)	Experimental group (EG) (*n*=40)	*χ*^2^/t	*P* value
Gender [*n* (%)]	Male	10 (25.0)	12 (30.0)	0.251	0.617
	Female	30 (75.0)	28 (70.0)		
Age (years)		37.17 ± 5.80	36.68 ± 5.75	1.042	0.301
Surgical method [*n* (%)]	SG	37 (92.5)	35 (87.5)	0.556	0.456
	RYGB	3 (7.5)	5 (12.5)		
BMI (kg/m^2^)		37.01 ± 3.82	36.31 ± 4.15	0.349	0.728
Education level [*n* (%)]	High school and above	30 (75.0)	27 (67.5)	0.549	0.459
	Below high school	10 (25.0)	13 (32.5)		

**Figure 1:**
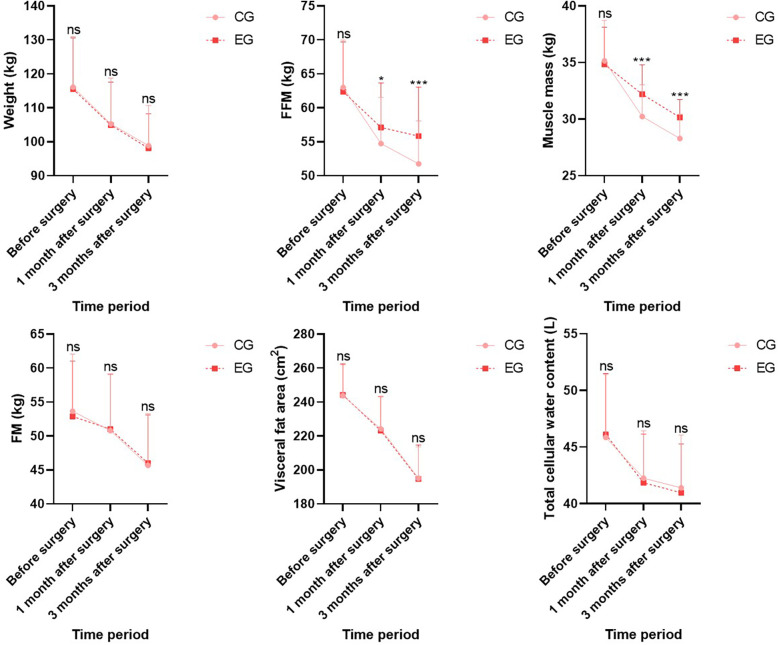
Body composition indicators in both groups. Versus CG, ns, no significance, *P < 0.05, ***P < 0.001.

Before surgery, no statistical significance in weight, FFM, muscle mass, FM, visceral fat area, and total cellular water content demonstrated between both groups (*P* > 0.05). One month and three months after surgery, weight, FFM, muscle mass, FM, visceral fat area, and total cellular water content in both groups demonstrated depletion relative to those before surgery (*P* < 0.05), whereas FFM and muscle mass in EG demonstrated elevation relative to those in CG during the same period, indicating statistical significance (*P* < 0.05); no statistical significance in weight, FM, visceral fat area, and total cellular water content demonstrated between both groups during the same period (*P* > 0.05; Figure 1).

### Comparison of inflammatory and blood glucose indicators between both groups

**Figure 2:**
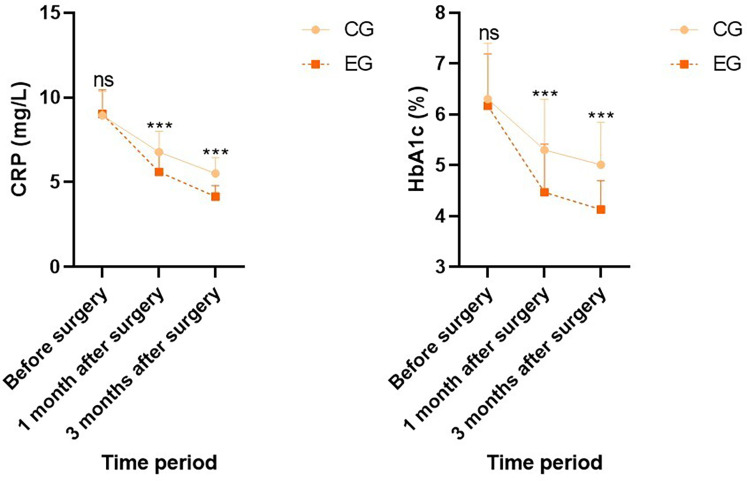
Inflammatory and blood glucose indicators in both groups. Versus CG, ns, no significance, ***P < 0.001.

Before surgery, no statistical significance in CRP and HbA1c levels demonstrated between both groups (*P* > 0.05). One month and three months after surgery, CRP and HbA1c levels in both groups demonstrated depletion relative to those before surgery (*P* < 0.05), and CRP and HbA1c levels in EG demonstrated depletion relative to those in CG during the same period, indicating statistical significance (*P* < 0.05; Figure 2).

### Comparison of nutritional indicators between both groups

**Figure 3:**
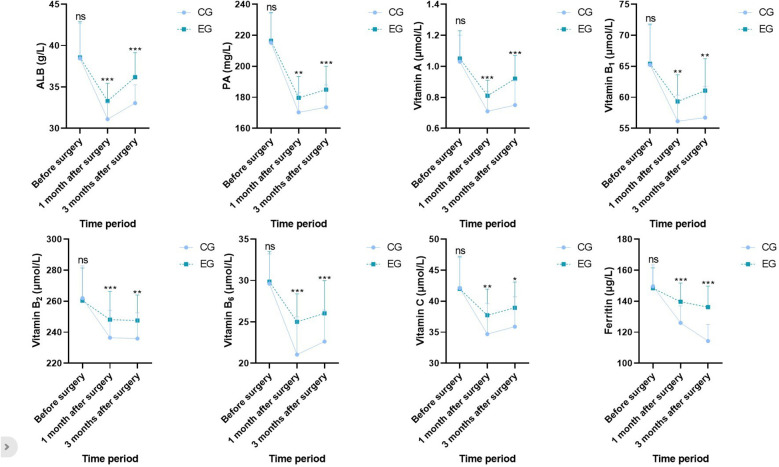
Nutritional indicators in both groups. Versus CG, ns, no significance, *P < 0.05, **P < 0.01, ***P < 0.001.

Before surgery, no statistical significance in serum ALB, PA, vitamin A, vitamin B_1_, vitamin B_2_, vitamin B_6_, vitamin C, and ferritin levels demonstrated between both groups (*P* > 0.05). One month and three months after surgery, ALB, PA, vitamin A, vitamin B_1_, vitamin B_2_, vitamin B_6_, vitamin C, and ferritin levels in both groups demonstrated depletion relative to those before surgery (*P* < 0.05), whereas ALB, PA, vitamin A, vitamin B_1_, vitamin B_2_, vitamin B_6_, vitamin C, and ferritin levels in EG demonstrated elevation relative to those in CG during the same period, indicating statistical significance (*P* < 0.05; Figure 3).

### Comparison of negative emotion scores between both groups

**Figure 4:**
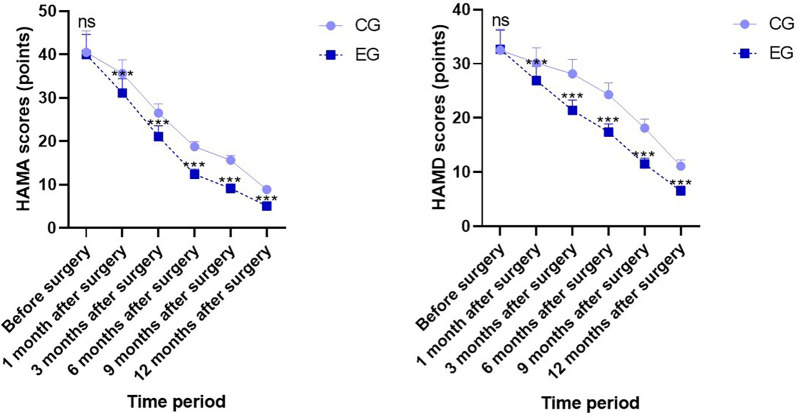
Negative emotion scores in both groups. Versus CG, ns, no significance, ***P < 0.001.

Before surgery, no statistical significance in HAMA and HAMD scores demonstrated between both groups (*P* > 0.05). One month, three months, six months, nine months, and twelve months after surgery, HAMA and HAMD scores in both groups demonstrated depletion relative to those before surgery, and HAMA and HAMD scores in EG demonstrated depletion relative to those in CG during the same period, indicating statistical significance (*P* < 0.05; Figure 4).

## Discussion

Obesity is a disease with excess energy and one of the major reasons for lifestyle related diseases (such as DM, non-alcoholic fatty liver disease and cardiovascular diseases) (21). MBS is widely recognized as the most clinically valuable and cost-effective treatment for moderate to severe obesity.

Currently, MBS is mostly carried out with participation of multiple disciplines, among which nutrition intervention is an essential part. So far, there have been few reports in China focusing on changes in muscle and adipose tissue of patients undergoing MBS under nutrition intervention, which have close relation to weight loss effect. An energy-limited and high-protein diet is a feasible medical nutrition treatment plan after surgery, which can downregulate postoperative respiratory quotient level, reduce muscle mass loss, delay FFM loss, and attenuate decline rate of basal metabolic rate, achieving satisfactory weight loss effect (22, 23). The bioelectrical impedance principle to determine body composition has become a widely applied and easily implementable tool in clinical practice, and its measurement values are basically consistent with those obtained by dual energy x-ray absorption method, which can receive accurate application for evaluating body composition after MBS. Herein, after surgery, weight, FFM, muscle mass, FM, visceral fat area, and total cellular water content in both groups demonstrated depletion relative to those before surgery, whereas FFM and muscle mass in EG demonstrated elevation relative to those in CG during the same period. This indicates that loss of postoperative FFM has relation to decrease in postoperative protein intake, and supplementing protein through nutrition intervention can repress loss of FFM. Thus, if patients’ appetite decreases after surgery, they should receive encouragement to supplement protein according to their actual needs. Furthermore, CRP and HbA1c levels in both groups depicted a decreasing trend after surgery, and CRP and HbA1c levels in EG demonstrated depletion relative to those in CG during the same period, indicating that nutrition intervention can ameliorate inflammatory status and blood glucose level of the body.

Serum protein levels are the most commonly applied indicators reflecting nutritional status of patients, including ALB, PA, etc (24). Herein, after surgery, ALB and PA levels in both groups demonstrated depletion relative to those before surgery, whereas ALB and PA levels in EG demonstrated elevation relative to those in CG during the same period. This indicates that nutrition intervention can effectively improve postoperative nutritional status of patients. Multiple patients undergoing MBS often lack several essential vitamins and microelements for metabolism before surgery. For instance, obese patients often experience iron deficiency before surgery due to chronic inflammation, which stimulates synthesis of ferritin and affects absorption of iron in the body (25); elevated intake of high-fat foods can lead to a deficiency of various vitamins such as folic acid, vitamin A, and vitamin C (26); elevation in body fat can reduce lipid-soluble vitamins in serum, such as vitamins A, D, E, and K (27). MBS may lead to or exacerbate micronutrient deficiencies (28). Different types of MBS have varying degrees of impact on absorption of micronutrients, and restrictive surgery (such as SG) exerts a smaller impact on absorption of essential micronutrients relative to malabsorptive surgery (such as RYGB). The occurrence of micronutrient deficiency after MBS often leads to serious complications such as anemia, hair loss, and Wernicke's encephalopathy (29). Herein, after surgery, vitamin A, vitamin B_1_, vitamin B_2_, vitamin B_6_, vitamin C, and ferritin levels in both groups demonstrated depletion relative to those before surgery, whereas vitamin A, vitamin B_1_, vitamin B_2_, vitamin B_6_, vitamin C, and ferritin levels in EG demonstrated elevation relative to those in CG during the same period. This indicates that nutrition intervention can alleviate deficiency of microelements in patients. Nevertheless, further prospective studies are needed to determine major reasons for micronutrient deficiency in obese patients and specific strategies for postoperative nutritional supplementation.

Research indicates that approximately 42% of MBS candidates are diagnosed with mental disorders, with depression, anxiety, and eating disorders being particularly prevalent. However, following MBS surgery, the lack of specialized personnel and professional teams for long-term follow-up and management makes it challenging to intervene and assist patients with postoperative mental health. Increasing evidence suggests that continuous care under the IMB model extends mental health support from the hospital to the home, providing patients with stable and professional assistance. This study found that post-surgery, both groups exhibited depletion in HAMA and HAMD scores relative to pre-surgery levels. Compared to the CG group during the same period, the OG group demonstrated lower HAMA and HAMD scores. This indicates that continuous care under the IMB model effectively alleviates negative emotions such as anxiety and depression in MBS patients. This aligns with the psychological support role the IMB model plays in other conditions. Continuous care under the IMB model extends from hospital to home, guiding patients to adjust their mindset when facing stress during weight loss, reducing surrender response methods, and helping improve postoperative mental health. Additionally, promptly correcting deficiencies in key nutrients such as iron, vitamin B12, and vitamin D after surgery not only prevents anemia and metabolic abnormalities but may also directly improve mood and cognitive function by influencing neurotransmitters and neural function (30). Simultaneously, the physiological improvements resulting from effective nutritional management can enhance patients’ self-efficacy and body image, thereby alleviating anxiety and depression. Therefore, the psychological improvements observed in this study are likely the synergistic outcome of professional psychological support combined with optimized physiological nutritional status.

This study has the following limitations: 1. It was conducted at a single medical center with a relatively small sample size (*n* = 80). This limits the generalizability of the findings to a broader population, such as patients from different regions or healthcare systems. The small sample size may also have reduced the ability to detect clinically significant differences between groups in certain secondary outcomes. 2. The follow-up period was relatively short. Key endpoint assessments, particularly for body composition and biochemical nutritional indicators, were conducted only at 1 month and 3 months postoperatively. This short follow-up duration does not allow for evaluation of the long-term effects of the nursing intervention model. Similarly, psychological assessments were limited to within 3 months, which is insufficient to fully determine the lasting impact of the intervention on patients’ anxiety and depression. 3. Heterogeneity in surgical methods and lack of stratified analysis. The study included different surgical procedures, SG and RYGB. These two surgeries differ significantly in their effects on digestive absorption mechanisms and nutritional metabolism. The study did not stratify randomization based on surgical type, which may have introduced reporting or selection bias and confounded the true effects of the intervention.

This study validated a structured, replicable postoperative management protocol. Integrating the “Information-Motivation-Behavioral Skills” model with proactive nutritional interventions effectively improves both physiological and psychological outcomes in bariatric surgery patients. Furthermore, the intervention strategies provided herein serve as a practical template for other postoperative follow-up systems, contributing to enhanced overall care quality—particularly in preserving muscle mass, improving nutritional status, and alleviating psychological factors. However, our findings require further validation in larger, more rigorously designed studies to address existing limitations. This includes conducting multicenter, large-sample randomized controlled trials and extending follow-up periods to assess the long-term efficacy of this model. The findings of this study also provide clear guidance for nursing practices following metabolic and bariatric surgery (MBS). A nurse-led MBS postoperative management team exercising autonomy in postoperative care and monitoring can effectively improve patients’ long-term quality of life, including nutritional status, body composition, and mental health.

## Conclusion

Combination of continuous nursing under IMB mode and nutrition intervention can mitigate anxiety and depression of patients after MBS, consolidate surgical effect, effectively reduce loss extent of FFM, improve postoperative nutritional status of patients, and alleviate deficiency of microelements, thereby gradually changing people's understanding and acceptance of MBS, which have positive significance for future development of MBS.

## Data Availability

The raw data supporting the conclusions of this article will be made available by the authors, without undue reservation.
